# Monolithic CAD/CAM Implant-Retained Overdentures: A Solution for Limited Restorative Space

**DOI:** 10.1155/crid/9034722

**Published:** 2025-02-15

**Authors:** Abdulrahman Almalki

**Affiliations:** Department of Prosthetic Dental Science, Prince Sattam bin Abdulaziz University, Al-kharj, Saudi Arabia

**Keywords:** attachments, CAD/CAM, dental implants, digital overdentures, restorative space

## Abstract

This case report applies computer-aided design and computer-aided design manufacturing (CAD/CAM) technology in fabricating an implant-retained monolithic overdenture for a patient with limited vertical restorative space. Traditionally, a vertical space of 9 to 12 mm is required for conventional overdentures; however, this patient presented with only 6 mm available. Through the integration of CAD/CAM, a novel solution was designed that optimized both function and esthetics within the constrained space.

## 1. Introduction

Implant-retained overdentures have become increasingly popular due to their cost-effectiveness and the potential to serve as transitional prostheses, eventually leading to implant-supported fixed solutions [1, 2]. A critical determinant for the success of implant-retained overdentures is the adequacy of restorative space, which is imperative for achieving functional stability and esthetic excellence [3]. Traditionally, the fabrication of conventional overdentures necessitates a vertical space ranging from 9 to 12 mm [4, 5]. This requirement becomes a significant challenge when faced with limited restorative space.

The introduction of computer-aided design and computer-aided manufacturing (CAD/CAM) technologies has significantly developed in last the few years [6]. These technologies have facilitated the construction of complex and precise prostheses that were previously unattainable with traditional techniques [7, 8]. Monolithic dentures represent one of the many creative solutions made possible by CAD/CAM technologies, combining the denture base and teeth into a singular, cohesive block [9]. This approach not only enhances the prosthetic's strength and integration but also overcomes common complications associated with conventional dentures, such as fracture and debonding of teeth from the base [10, 11].

This case report describes clinical challenge of a patient presenting with extremely limited restorative space. Leveraging the capabilities of CAD/CAM technology, a monolithic overdenture was designed and fabricated to conform to the limited space available. The monolithic design was critical in overcoming the space limitation, offering a novel solution that integrated the denture teeth directly into the base, thus reducing the required vertical space without compromising on prosthetic integrity.

## 2. Clinical Report

A 45-year-old female was referred to our prosthodontic clinic at Signature Smile Dental Office, OH, United State for the fabrication of a maxillary implant overdenture after previous unsatisfactory attempts. The patient's medical history included well-controlled hypertension, with xerostomia noted as a possible medication side effect. Her dental history was significant for multiple extractions due to caries and unsuccessful endodontic treatments. The chief complaint was dissatisfaction with the esthetics of her interim denture and wax try-in provided by her previous dentist, as she desires a more natural appearance.

**Figure 1:**
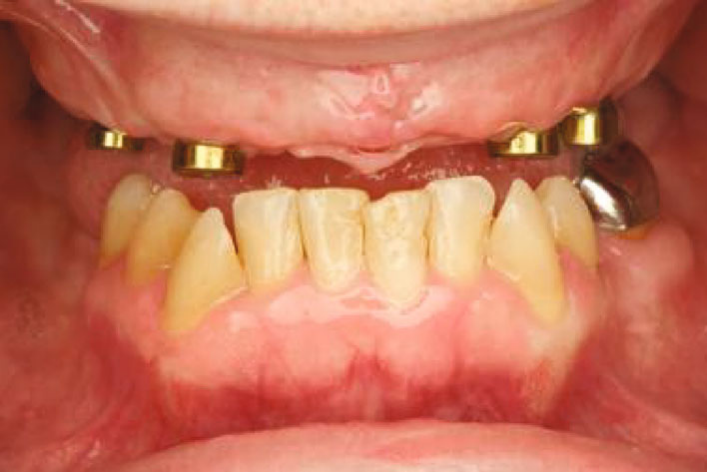
Pretreatment intraoral view in the approximate desired OVD.

**Figure 2:**
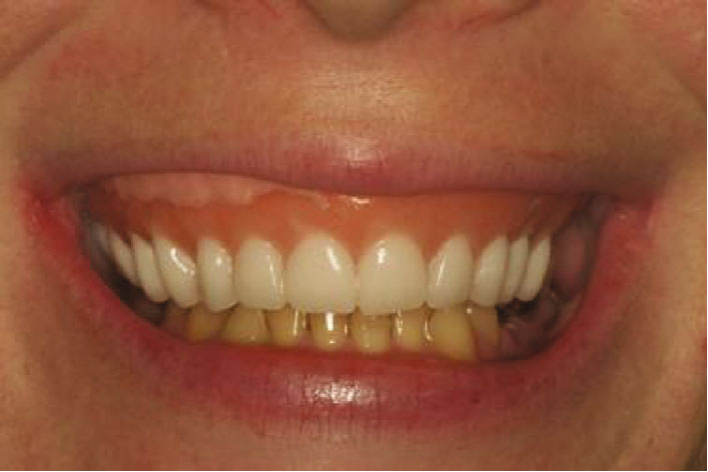
Maxillary interim prosthesis with excessive denture base display, short and misaligned teeth with visible reline material.

Comprehensive assessment and data acquisition revealed that the patient has four implants placed in the maxillary arch (Helix GM; Neodent) with challenging restorative space (Figure 1). This was compounded by the patient's high smile line and ambitious esthetic expectations. Evaluation of the existing maxillary interim denture revealed a pronounced display of denture base, improperly aligned, undersized denture teeth, coupled with an excessive occlusal vertical dimension (OVD) (Figure 2).

The relining material was meticulously removed from the existing interim denture, and the denture base and teeth were adjusted to establish an appropriate OVD. Afterward, approximately 2 mm of the denture base was reduced to accommodate impression materials. Final impression was made starting with border molding using heavy-body polyvinyl siloxane (Imprint 3; 3M ESPE), and a final impression was taken using a light-body polyvinyl siloxane (Imprint 3; 3M ESPE).

**Figure 3:**
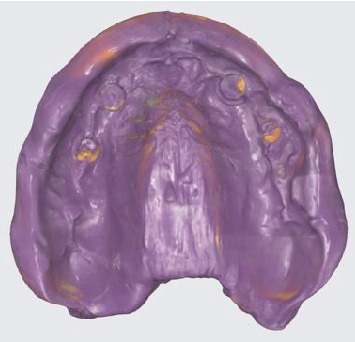
Complete scan of the final impression taken with the existing maxillary interim denture in position.

**Figure 4:**
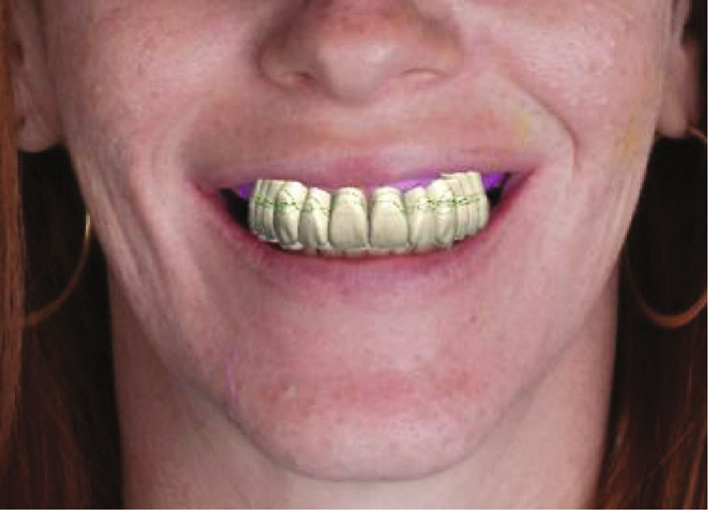
A digital design of a dental prosthesis created using 3Shape software, demonstrating a facially driven approach for optimal esthetics and function.

**Figure 5:**
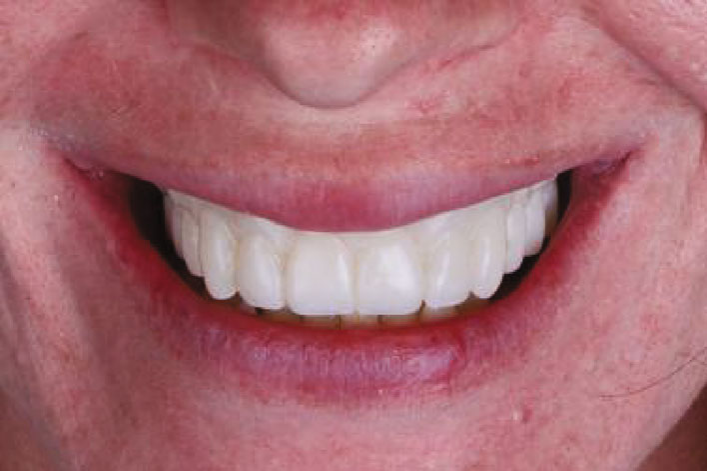
A 3D- printed dental prototype to evaluate esthetics, phonetics, and occlusal vertical dimension (OVD) in the patient's smile.

A complete scan of the existing denture after the impression was made using an intraoral scanner (Medit i700; Medit); the intaglio, polished, and teeth surfaces were scanned (Figure 3), followed by the opposing mandibular arch and bite registration captured at the predetermined OVD. The resultant stereolithography (STL) files were imported into 3Shape CAD software (3Shape Dental System; 3Shape) for the digital design of the monolithic denture (Ivotion; Ivoclar Vivadent) (Figure 4). Following the Ivotion workflow, a new setup was created using a preset tooth library within the system. Prior to milling, a prototype was 3D printed for intraoral evaluation to ensure satisfactory teeth display, OVD, and esthetics, receiving patient approval (Figure 5).

**Figure 6:**
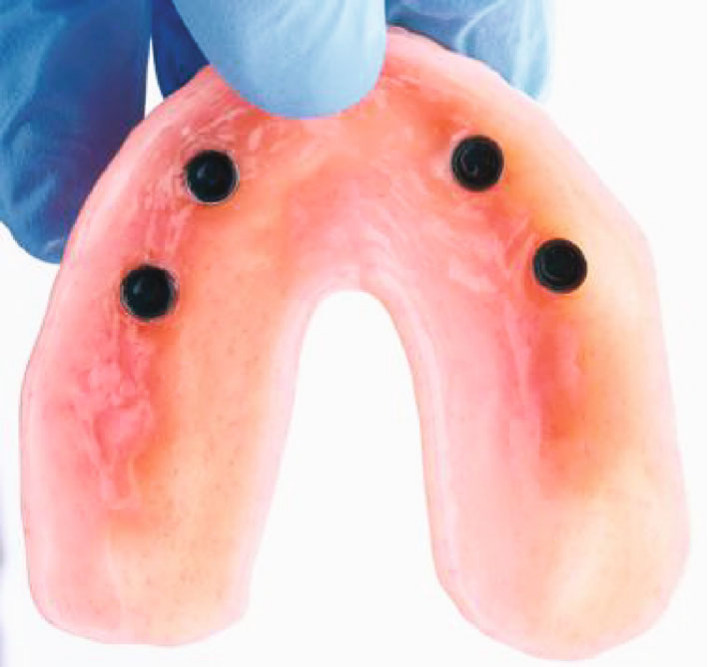
Intaglio surface of a palateless maxillary overdenture after chairside processing of the attachment metal housings.

During the insertion appointment, prosthesis adaptation, tissue fit, and occlusion were examined using an articulator and shim stock foil to confirm heavy posterior contacts and anterior disclusion in centric occlusion, with bilateral balance in excursive movements. Locator attachments were placed according to the manufacturer's specifications, with radiographic verification of passive fit. The metal housings were then chairside connected with autopolymerizing resin. Post insertion instructions including maintenance were given and demonstrated to the patient (Figure 6).

**Figure 7:**
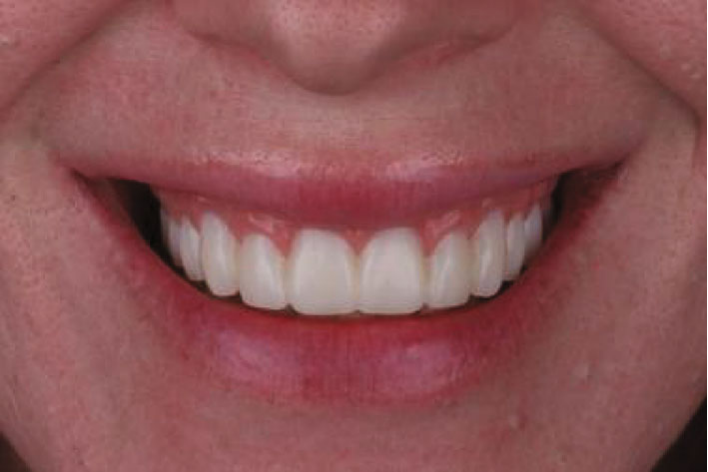
Posttreatment smile with the definitive prosthesis.

**Figure 8:**
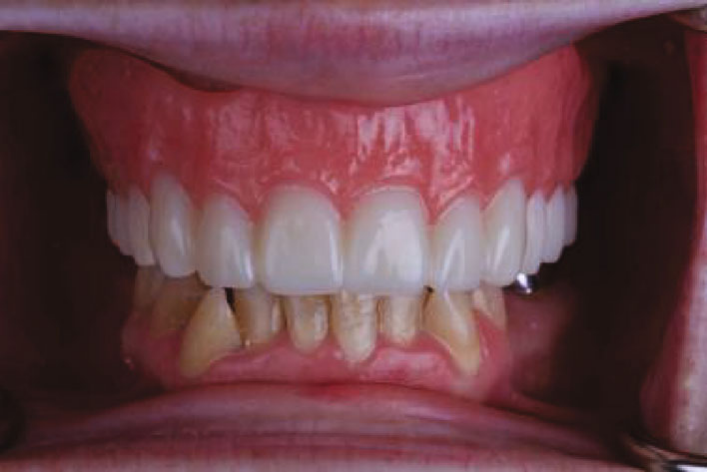
One- year follow-up displaying the integrity of the definitive prosthesis material and maintained esthetics.

The definitive prosthesis met both esthetic and functional objectives, consistent with the patient's expectations (Figure 7). The patient was educated on maintenance and the risks of peri-implant diseases. Follow-up at 12 months posttreatment indicated successful integration and patient satisfaction without biological or prosthetic complications (Figure 8).

## 3. Discussion

This case report demonstrates the capabilities of CAD/CAM technology in managing challenging implant overdentures with restricted restorative space. Limited vertical restorative space often necessitated a compromise between prosthetic function and esthetics. However, in the case presented, CAD/CAM technology facilitated the creation of a monolithic overdenture that satisfied the clinical needs and provided satisfactory esthetic and function outcomes.

Although various design options exist for maxillary implant overdentures, the palateless design stands out as particularly advantageous [12]. This design enhances patient comfort, preserves natural oral sensations including taste, and is exceptionally beneficial for patients with severe gag reflexes or anatomical variations such as large tori or bony exostoses [13, 14]. The primary concern with a palateless overdenture is the potential for decreased retention. Nonetheless, many studies suggest that four implants can effectively compensate for the removal of the palatal coverage, thereby maintaining prosthesis retention without compromising the integrity or functionality of the denture [15, 16].

Polymethyl methacrylate (PMMA) processed through conventional methods has been traditionally associated with suboptimal mechanical strength, particularly at minimal thickness [17], leading to a risk of fracture [18]. To overcome this risk, the reinforcement of implant overdentures with a metal substructure has frequently been advocated [19, 20]. Nonetheless, advancements in milling technology have introduced PMMA with enhanced physical properties surpassing those of conventionally processed materials [11]. As a result, milled CAD/CAM PMMA could be used with no need for additional reinforcement, marking a critical transition toward the cost-efficient fabrication of high-quality implant overdentures. In the current case, a 1-year follow-up indicated that the prosthesis maintained its integrity, underscoring the potential of milled PMMA in clinical applications.

Wear of denture teeth is one of the most common complications observed in both conventional dentures and implant-supported prostheses [20]. Excessive wear of acrylic teeth can lead to occlusal interferences during excursive movements, potentially compromising the stability and retention of the prosthesis [21]. To address this issue, several strategies have been employed to enhance the wear resistance of acrylic teeth, including the incorporation of double-cross-linked (DCL) agents into PMMA resin and the use of nanocomposite (NC)-infused resin teeth with reinforced facial and incisal dentin layers [22]. A recent study comparing the wear resistance of DCL, NC, and milled PMMA teeth demonstrated that milled PMMA exhibited superior wear resistance compared to NC and performed comparably to DCL teeth [23]. In the present case, despite the presence of opposing natural dentition, the amount of wear observed in the milled denture was found to be insignificant, aligning with the reported adequate wear resistance of milled acrylic teeth.

The favorable short-term outcomes of this case may suggest a paradigm shift in design strategies for implant overdentures, particularly when dealing with limited restorative space. This method underscores the value of a patient-centered approach, ensuring that treatment objectives are in harmony with the patient's comfort and expectations. Additionally, it demonstrates the efficiency of streamlining the treatment process, ultimately reducing the number of visits required, which can significantly enhance patient experience.

## 4. Summary

The presented case report details a successful clinical application of a monolithic CAD/CAM-fabricated overdenture in a patient with significantly limited restorative space. This approach has demonstrated that with technological advancements, effective solutions that do not compromise esthetic or functional outcomes are achievable, even in complex cases. This case also reinforces the need for interdisciplinary collaboration in planning and executing complex dental treatments.

## Data Availability

Data is available on request.

## References

[1] Roy S., Maji S., Paul R., Bhattacharyya J., Goel P. (2020). A comparison of cost and cost-effectiveness analysis of two- implant-retained overdentures versus other removable prosthodontic treatment options for edentulous mandible: a systematic review. *The Journal of Indian Prosthodontic Society*.

[2] Takanashi Y., Penrod J. R., Lund J. P., Feine J. S. (2004). A cost comparison of mandibular two-implant overdenture and conventional denture treatment. *The International Journal of Prosthodontics*.

[3] Ahuja S., Cagna D. R. (2011). Classification and management of restorative space in edentulous implant overdenture patients. *The Journal of Prosthetic Dentistry*.

[4] Ahuja S., Cagna D. R. (2010). Defining available restorative space for implant overdentures. *The Journal of Prosthetic Dentistry*.

[5] Carpentieri J., Greenstein G., Cavallaro J. (2019). Hierarchy of restorative space required for different types of dental implant prostheses. *Journal of the American Dental Association (1939)*.

[6] Wang C., Shi Y.-F., Xie P.-J., Wu J.-H. (2021). Accuracy of digital complete dentures: a systematic review of in vitro studies. *The Journal of Prosthetic Dentistry*.

[7] Almalki A., Sourvanos D., Kutkut N. (2023). Facially driven digital workflow for maxillary and mandibular milled implant-retained overdentures using two different unsplinted attachment systems: case report. *The Compendium of Continuing Education in Dentistry*.

[8] Maragliano-Muniz P., Kukucka E. D. (2021). Incorporating digital dentures into clinical practice: flexible workflows and improved clinical outcomes. *Journal of Prosthodontics*.

[9] Bidra A. S., Farrell K., Burnham D., Dhingra A., Taylor T. D., Kuo C.-L. (2016). Prospective cohort pilot study of 2-visit CAD/CAM monolithic complete dentures and implant-retained overdentures: clinical and patient-centered outcomes. *The Journal of Prosthetic Dentistry*.

[10] Goodacre B. J., Goodacre C. J., Baba N. Z., Kattadiyil M. T. (2018). Comparison of denture tooth movement between CAD-CAM and conventional fabrication techniques. *The Journal of Prosthetic Dentistry*.

[11] Baba N. Z., Goodacre B. J., Goodacre C. J., Müller F., Wagner S. (2021). CAD/CAM complete denture systems and physical properties: a review of the literature. *Journal of Prosthodontics*.

[12] Mericske-Stern R. (1998). Treatment outcomes with implant-supported overdentures: clinical considerations. *The Journal of Prosthetic Dentistry*.

[13] Anadioti E., Gates W. D., Elpers J., de Kok I. J., Cooper L. F. (2019). Retrospective cohort analysis of maxillary overdentures retained by unsplinted implants. *The Journal of Prosthetic Dentistry*.

[14] Kutkut A., Bertoli E., Frazer R., Pinto-Sinai G., Fuentealba Hidalgo R., Studts J. (2018). A systematic review of studies comparing conventional complete denture and implant retained overdenture. *Journal of Prosthodontic Research*.

[15] Damghani S., Masri R., Driscoll C. F., Romberg E. (2012). The effect of number and distribution of unsplinted maxillary implants on the load transfer in implant-retained maxillary overdentures: an in vitro study. *The Journal of Prosthetic Dentistry*.

[16] Sadowsky S. J. (2007). Treatment considerations for maxillary implant overdentures: a systematic review. *The Journal of Prosthetic Dentistry*.

[17] Zafar M. S. (2020). Prosthodontic applications of polymethyl methacrylate (PMMA): an update. *Polymers*.

[18] Andreiotelli M., Att W., Strub J.-R. (2010). Prosthodontic complications with implant overdentures: a systematic literature review. *The International Journal of Prosthodontics*.

[19] Rodrigues A. H. C. (2000). Metal reinforcement for implant-supported mandibular overdentures. *The Journal of Prosthetic Dentistry*.

[20] Goodacre B. J., Goodacre S. E., Goodacre C. J. (2018). Prosthetic complications with implant prostheses (2001-2017). *European Journal of Oral Implantology*.

[21] Abdulhameed N., Volschow B., Abedi T. (2023). Clinical wear of different types of denture teeth after one year in service: a clinical study. *The Journal of Prosthetic Dentistry*.

[22] von Fraunhofer J. A., Razavi R., Khan Z. (1988). Wear characteristics of high-strength denture teeth. *The Journal of Prosthetic Dentistry*.

[23] Kim S. T., Cook D. R., Albouy J.-P., De Kok I., Sulaiman T. A. (2022). Linear and volumetric wear of conventional and milled denture teeth. *Journal of Esthetic and Restorative Dentistry*.

